# OGTT 1h serum C-peptide to plasma glucose concentration ratio is more related to beta cell function and diabetes mellitus

**DOI:** 10.18632/oncotarget.15239

**Published:** 2017-02-09

**Authors:** Hongmei Zhang, Bingxian Bian, Fan Hu, Qing Su

**Affiliations:** ^1^ Department of Endocrinology, Xinhua Hospital Affiliated to Shanghai Jiao Tong University School of Medicine, Shanghai, China; ^2^ Department of Clinical Laboratory, Xinhua Hospital Affiliated to Shanghai Jiao Tong University School of Medicine, Shanghai, China

**Keywords:** oral glucose tolerance test, C-peptide index, disposition index, β-cell function, diabetes mellitus

## Abstract

**Objective:**

To clarify the association between C-peptide index and pancreatic beta cell function and diabetes.

**Materials and methods:**

We carried out a retrospective analysis of 1021 patients aged 27 to 80 without diabetes from January 2012 to January 2016. All subjects underwent a 75-g oral glucose tolerance test. Blood samples were drawn at 0, 30, 60, 120 and 180 min after the glucose load. Plasma glucose concentrations, serum insulin levels, C-peptide levels, hemoglobin A1c (HbA1c), and other biochemical indicators were determined. C-peptide index was calculated as the ratio of C-peptide to plasma glucose. Disposition index was calculated as the result of the insulin sensitivity × insulin secretion. Area under the receiver operating characteristic curve was used to compare the diagnostic ability of C-peptide index for type 2 diabetes.

**Results:**

C-peptide index 1h was the most related one to disposition index (*r* = 647, p<0.001) and C-peptide release (*r* = 0.879, *p*<0.001). Both C-peptide index 1h (Exp(β) = 0.28, *p*<0.001) and 2h (Exp(β) = 0.42, *p*<0.001) were independently associated with disposition index, but the OR of C-peptide index 1h for diabetes was much lower. Area under the receiver operating characteristic curve of both C-peptide index 1h and 2h were all above 0.9, but the area of C-peptide index 1h was the highest one (0.937 *vs* 0.917). C-peptide index 1h has the highest diagnostic value (sensitivity = 90%, specificy = 85.2% *vs* sensitivity = 83.5%, specificy = 87.9%).

**Conclusion:**

C-peptide index after oral glucose ingestion may reflect the maximal β-cell function and is more related to diabetes. C-peptide index 1h is the most relevant one.

## INTRODUCTION

Progressive pancreatic β-cell function failure and insulin resistance are the key characteristics of type 2 diabetes mellitus (T2DM). Once the excessive secretion of insulin can no longer compensate for the degree of insulin resistance, clinically significant hyperglycemia will happen. Insulin secreted from pancreatic β-cells will be partially cleared in the liver before entering the peripheral circulation [1, 2]. The concentration of insulin calculated in peripheral blood can't represent the total amount of insulin secreted by the pancreas. On the contrary, C-peptide secreted with insulin in equimolar amounts is not cleared in the liver. The peripheral plasma C-peptide concentrations reflect endogenous insulin secretion more accurately than serum insulin [3]. Moreover, C-peptide is capable of assessing beta cell function even in patients under insulin therapy [4].

The C-peptide index (CPI), a ratio of serum C-peptide to plasma glucose concentrations, is a readily measured index of β-cell function [4, 5]. It strongly correlates with β-cell area as estimated by histological analysis of surgical specimens of the human pancreas [6, 7]. Compared with fasting C-peptide level, postprandial C-peptide level is more capable of representing the maximal insulin secretory capacity, especially in patients with diabetes [8]. Postprandial CPI was a superior predictor and may be a more practical index to evaluate β-cell functional capacity compared with fasting CPI [4]. Although postprandial CPI appears to reveal the maximal β-cell functional capacity more precisely, the timing of sampling (i.e., 0.5h, 1h or 2 h after a meal) may affect postprandial CPI. Different postprandial CPI at different time points may be related to pancreatic β-cell function differently.

The disposition index (DI), initially developed with data acquired from an IVGTT [9] and lately calculated with data obtained from an OGTT [10, 11], is an index of β-cell function adjusted for insulin sensitivity. β-cells act in response to an augmentation in glucose with an augmentation in insulin, and this response is modulated by the severity of insulin resistance. Thus, the gold standard for β-cell function is DI [12]. In the present study, we are going to use OGTT data obtained from 1021 subjects to investigate at which time point the postprandial CPI is most closely related to DI and diabetes mellitus.

## MATERIALS AND METHODS

### Study participants

This is a retrospective study. The participants were from the clinic of Endocrinology of Xinhua Hospital Affiliated to Shanghai Jiaotong University School of Medicine between January 2012 and January 2016. We included 1021 patients (489 men and 532 women) in total. All of the 1021 patients were non-diabetic and aged 27 to 80 years old. They all underwent a 75-g oral glucose tolerance test (OGTT) to determine whether they were suffering from diabetes according to WHO 1999 diagnostic criteria. Height and body weight were measured at the clinic, body mass index (BMI) was calculated as body weight in kilograms divided by height in meters squared. The research protocol was approved by the Ethics Committee of Xinhua Hospital Affiliated to Shanghai Jiaotong University School of Medicine.

### Oral glucose tolerance test

After at least 10 h of overnight fasting, participants were given a standard 75-g glucose solution. Blood samples were drawn at 0, 30, 60, 120 and 180 min after the glucose load to determine glucose concentrations, serum insulin levels, C-peptide levels, hemoglobin A1c (HbA1c), lipids profile including triglycerides, high-density lipoprotein cholesterol, low-density lipoprotein cholesterol, alanine aminotransferase, aspartate aminotransferase, and creatinine.

### Laboratory determinations

All blood indices were tested by the laboratory of the Xinhua Hospital Affiliated to Shanghai Jiaotong University School of Medicine. Plasma glucose was measured by enzymatic hexokinase hotometric assay (Hitachi 7600, Tokyo, Japan). Insulin was determined by Luminescent immunoassays (ADVIA Centaur XP, Siemens, Berlin, German). C-peptide was determined by Luminescent immunoassays (Roche e601, Leverkusen, Germany). HbA1c was determined by high-performance liquid chromatography (BIO-RAD VARIANT II, California, USA). Serum alanine aminotransferase, aspartate aminotransferase, creatinine, triglycerides, total cholesterol, low-density lipoprotein cholesterol, high-density lipoprotein cholesterol were measured with an autoanalyzer (Hitachi 7600, Tokyo, Japan).

### Calculations

CPI was calculated as: 100 × serum C-peptide level (ng/mL)/plasma glucose level (mg/dL). Total C-peptide release was calculated using the ratio of total C-peptide AUC and total glucose AUC during 0-180 min of the OGTT (C-pepAUC180/GluAUC180). The Matsuda insulin sensitivity index (ISIM) includes both hepatic and muscle components of insulin resistance and is associated well with euglycaemic insulin clamp, it can be regarded as a measure of whole-body insulin sensitivity [13, 14]. DI is calculated as the result of the insulin sensitivity × insulin secretion as reported in other research: total disposition index DI180 = [InsAUC180/GluAUC180] × ISIM [12, 15, 16].

### Statistical analysis

Data were expressed as means ± SD. Differences between groups were tested by unpaired Student's *t*-test. Logistic regression analysis was used to examine the association of all the parameters and diabetes. Logistic regression models were used to estimate the odds ratios (ORs) and confidence intervals (CIs) for diabetes mellitus for every CPI. Potential confounding variables including age, gender, BMI, HbA1c were adjusted in the regression models. Correlation coefficients between the indices were calculated by Pearson correlation analysis. Multiple stepwise linear regression analysis was used to determine the relationship between different variables and DI. To compare the ability for predicting type 2 diabetes, we used area under the receiver operating characteristic curve (AROC). All statistical analysis was performed with the SPSS Statistical Package (version 13.0; SPSS Inc., Chicago, IL). *P* values < 0.05 were considered statistically significant.

## RESULTS

**Figure 1:**
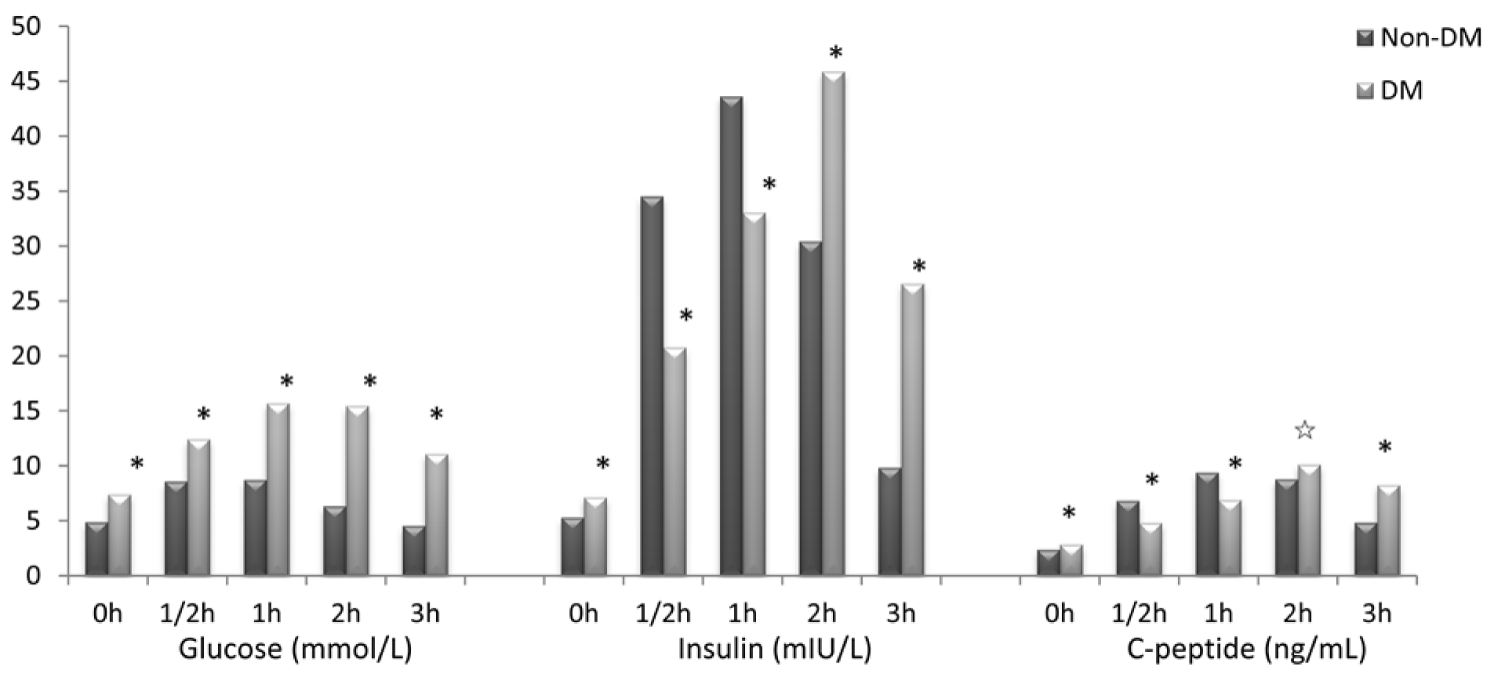
Blood glucose, serum insulin and serum C-peptide levels in DM and non-DM group *p < 0.01, ^☆^ *p* < 0.05.

Of all the 1021 studied subjects, 324 were diagnosed with diabetes after OGTT according to WHO 1999 diagnostic criteria (191 men and 133 women). 697 were non-diabetes (297 men and 400 women). The clinical characteristics of the subjects are shown in Table 1. When compared with those without DM, the subjects with DM showed significant differences in several clinical and laboratory characteristics including age, BMI, blood glucose indices, serum insulin levels and C-peptide levels. (see Table 1 and Figure 1).

**Table 1:** Clinical and laboratory characteristics of study subjects

Characteristics^a^	DM	Non-DM	*P* value
N	324	697	
Age (yr)^b^	59.97±13.58	52.56±17.89	<0.001
Sex (Male/Female)^b^	191/133	297/400	0.983
BMI (kg/m^2^)	24.38±3.36	23.62±3.17	<0.01
HbA1C (%)	7.48±1.77	5.76±0.50	<0.001
LDL (mmol/l)	2.87±0.95	2.82±0.79	0.844
HDL (mmol/l)	1.34±0.57	1.39±0.38	0.137
TG (mmol/l)	2.24±0.20	2.18±0.76	0.882
CHO (mmol/l)	4.84±1.42	4.68±1.13	0.449
ALT	32.09±4.09	30.79±5.05	0.097
AST	33.09±5.24	23.04±2.02	0.02
Scr	73.09±16.44	72.28±14.85	0.404

^a^Data are means ± SD, number (percent); *P* value was calculated after adjustment for age, gender.

^b^Not adjusted for itself.

Correlation analysis showed a remarkable correlation between DI and CPI 1/2h (*r* = 0.526, *P* < 0.001), DI and CPI 1h (*r* = 647, *P* < 0.001), DI and CPI 2h (*r* = 0.529, *P* < 0.001), DI and CPI 3h (*r* = 0.179, *P* < 0.001), HbA1c and CPI 0h (*r* = -0.288, *P* < 0.001), HbA1c and CPI 1/2h (*r* = -0.518, *P* < 0.001), HbA1c and CPI 1h (*r* = -0.584, *P* < 0.001), HbA1c and CPI 2h (*r* = -0.593, *P* < 0.001), HbA1c and CPI 3h (*r* = -0.351, *P* < 0.001), C-pepAUC/GluAUC and CPI 0h (*r* = 0.428, *P* < 0.001), C-pepAUC/GluAUC and CPI 1/2h (*r* = 0.846, *P* < 0.001), C-pepAUC/GluAUC and CPI 1h (*r* = 0.879, *P* < 0.001), C-pepAUC/GluAUC and CPI 2h (*r* = 832, *P* < 0.001), C-pepAUC/GluAUC and CPI 3h (*r* = 0.652, *P* < 0.001) after adjusted for age, gender, and BMI (see Table 2). A multiple regression analysis with a stepwise model was used to assess the independent variables that may affect DI levels. The main determinants of DI are CPI 1h (β = 0.652, *P* < 0.001), CPI 2h (β = 0.162, *p* = 0.02), HbA1c (β = -0.299, *P* < 0.001), and TG (β = -0.147, *P* < 0.001) (see Table 3). CPI 1h was shown to be the most relevant one to DI and C-peptide release.

**Table 2:** Crude and partial correlation between CPI at different time points and DI, HbA1c, and AUCc/AUCg

Variable		Crude r	Partial r^†^
CPI 0h	DI HbA1c C-pepAUC/GluAUC	0.001 -0.283** 0.422**	0.016 -0.288** 0.428**
CPI 1/2h	DI HbA1c C-pepAUC/GluAUC	0.539** -0.525** 0.845**	0.526** -0.518** 0.846**
CPI 1h	DI HbA1c C-pepAUC/GluAUC	0.660** -0.593** 0.869**	0.647** -0.584** 0.879**
CPI 2h	DI HbA1c C-pepAUC/GluAUC	0.535** -0.597** 0.833**	0.529** -0.593** 0.832**
CPI 3h	DI HbA1c C-pepAUC/GluAUC	0.141** -0.317** 0.626**	0.179** -0.351** 0.652**

*p < 0.01, **p < 0.001, †adjusted for age, gender, and BMI.

**Table 3:** Multiple stepwise linear regression analysis showing variables independently associated with DI

Independent variables	Standardized β	t	*P* value
CPI 1h	0.652	9.347	<0.001
CPI 2h	0.162	2.340	0.02
HbA1c	-0.299	-4.614	<0.001
TG	-0.147	-2.732	<0.001

The analysis also included age, gender, CPI 0h, CPI 1/2h, CPI 3h, CHO, LDL, HDL, BMI which were all excluded in the final model.

Logistic regression analysis showed that after adjusted for age and gender, BMI, HbA1C, TG, CHO, LDL and HDL, CPI at different time points were all associated with DM, but CPI 1h was the most relevant one: CPI 0h (Exp(β) = 0.68, *P* < 0.001), CPI 1/2h (Exp(β) = 0.34, *P* < 0.001), CPI 1h (Exp(β) = 0.28, *P* < 0.001), CPI 2h (Exp(β) = 0.42, *P* < 0.001), CPI 3h (Exp(β) = 0.86, *P* < 0.001) (see Table 4).

**Table 4:** Adjusted ORs and 95% CIs for diabetes according to CPI at different time points

	CPI (Mean ± SD)			Model 1 ORs (95% CI) *P*		Model 2 ORs (95% CI) *P*	
	DM	Non-DM	*P*				
CPI 0h	2.23 ± 1.20	2.73 ± 1.44	<0.001	0.69 (0.56-0.85)	<0.001	0.68 (0.55-0.83)	<0.001
CPI 1/2h	2.16 ± 1.54	4.57 ± 1.97	<0.001	0.35 (0.27-0.45)	<0.001	0.34 (0.27-0.45)	<0.001
CPI 1h	2.51 ± 1.52	6.56 ± 2.63	<0.001	0.29 (0.22-0.38)	<0.001	0.28 (0.22-0.39)	<0.001
CPI 2h	3.55 ± 2.14	7.85 ± 2.68	<0.001	0.43 (0.37-0.51)	<0.001	0.42 (0.35-0.50)	<0.001
CPI 3h	4.64 ± 2.90	5.80 ± 2.40	<0.01	0.89 (0.82-0.97)	<0.01	0.86 (0.79-0.94)	<0.01

Model 1 unadjusted

Model 2 further adjusted for age and gender, BMI, HbA1C, TG, CHO, LDL, HDL.

In ROC curve analysis, we found that CPI 1h has the highest diagnostic value to predict diabetes mellitus (AROC = 0.937, sensitivity = 90%, specificy = 85.2%). Other results were as follows: CPI 0h (AROC = 0.628, sensitivity = 46.1%, specificy = 78.5%), CPI 1/2h (AROC = 0.878, sensitivity = 78.3%, specificy = 87.9%), CPI 2h (AROC = 0.917, sensitivity = 83.5%, specificy = 87.9%), CPI 3h (AROC = 0.655, sensitivity = 36.5%, specificy = 91.9%) (see Table 5).

**Table 5:** CPI at different time points and prediction of type 2 Diabetes

Variables	AROC	95% CI	Cut-off point	Sensitivity	specificity
CPI 0h	0.628	0.563-0.693	1.814	46.1%	78.5%
CPI 1/2h	0.878	0.836-0.921	2.595	78.3%	87.9%
CPI 1h	0.937	0.910-0.965	4.125	90.0%	85.2%
CPI 2h	0.917	0.883-0.950	5.254	83.5%	87.9%
CPI 3h	0.655	0.590-0.720	2.595	36.5%	91.9%

AROC, area under receiver operating characteristic curve

## DISCUSSION

C-peptide is widely accepted as a marker of beta cell function [17, 18], since it is not extracted by the liver like insulin. Glucose itself is a major stimulus of β-cells, insulin secretion is incremented by hyperglycemia seen in patients with diabetes. Thus, in order to precisely assess β-cell function, C-peptide level should be adjusted for glucose. The C-peptide index (CPI) refers to C-peptide immunoreactivity adjusted by plasma glucose level. Postprandial CPI is more closely related to β-cell function than fasting CPI. Some research indicates that the insulin requirement for the management of Type 2 diabetes is more strongly associated with postprandial CPI than with fasting CPI [4]. Postprandial CPI is the best predictive marker for the requirement of insulin therapy [8].

The disposition index (DI) is considered to reflect the insulin secretory capacity adjusted for insulin sensitivity and is regarded as a useful measure of true β-cell function. Some researchers recently reported that postprandial CPI, but not fasting CPI significantly associated with DI calculated by glucose clamp technique [19]. Other researchers also reported that compared with fasting CPI and HOMA-β, postprandial CPI displayed the strongest association with β-cell mass in surgical pancreatic specimens from patients [6]. Thus, postprandial CPI probably predicts the maximal β-cell secretory capacity and possibly more closely reflect the β-cell function [8]. Our findings are consistent with these results. We also found that postprandial CPI is more related to β-cell function. We found CPI 1h was the most related one to DI and to C-peptide release (Table 2). Both CPI 1h and CPI 2h were all independently associated with DI, but the OR of CPI 1h for diabetes was much lower (Table 3).

In a previous study [20], the authors found that oral DI calculated during OGTT is the best predictor for incidence of diabetes in the future in non-diabetic subjects. Oral DI and clamp DI were correlated only with postprandial CPI, not with fasting CPI, indicating that postprandial CPI takes into better account information associated not only to insulin secretion but also to insulin sensitivity [19]. Postprandial CPI was more closely related to future development of diabetes than fasting and 2-h glucose [21]. In the present study, we also found postprandial CPI are more related to diabetes compared with fasting CPI (Table 4). And CPI 1h is the most related one to diabetes. CPI 1h has the highest diagnostic value (Table 5).

The present study has several limitations. First, this study is not a prospective study, we can't draw a conclusion that CPI 1h is a predictor for diabetes. Based on our results, a prospective study is going ahead. Second, since the present study is retrospective, unidentified confounders might exist. Despite the existed limitations, it is still worth paying close attention to the results because the prevalence of diabetes increased globally, our results reflect the “real world” situation. CPI 1h can possibly be used as an index for β-cell function in practical field.

## CONCLUSIONS

The present study suggests that although the HOMA index might have some relevance to assessing the functional integrity of insulin secretion, it only represents the fasting state. Future studies should consider the use of a CPI after oral glucose ingestion as a functional estimate of β-cell function. Postprandial CPI is likely to better reflect the maximal β-cell function compared with the fasting CPI, and it is easily calculated using postprandial C-peptide and glucose levels measured at the diagnosis of type 2 diabetes. Postprandial CPI may add predictive utility to other predictors for future glycemic control and help to chose an optimal management for individual patient with type 2 diabetes [22]. Postprandial 1h CPI may be the most useful one.
